# Seroprevalence and associated factors with *Neospora caninum* infection in sheep and goats slaughtered in the state of Paraíba, Brazil

**DOI:** 10.1590/S1984-29612023068

**Published:** 2023-11-27

**Authors:** Samira Pereira Batista, Samara dos Santos Silva, Wlysse Ferreira Sarmento, Rinaldo Aparecido Mota, Thais Ferreira Feitosa, Vinícius Longo Ribeiro Vilela

**Affiliations:** 1 Programa de Pós-graduação em Ciência e Saúde Animal, Universidade Federal de Campina Grande – UFCG, Patos, PB, Brasil; 2 Departamento de Medicina Veterinária, Universidade Federal Rural de Pernambuco – UFRPE, Recife, PE, Brasil; 3 Departamento de Medicina Veterinária, Instituto Federal de da Paraíba – IFPB, Sousa, PB, Brasil

**Keywords:** Neosporosis, small ruminants, semi-arid, Neosporose, pequenos ruminantes, semiárido

## Abstract

The objective was to describe the seroprevalence of anti-*Neospora caninum* antibodies in goats and sheep slaughtered in the state of Paraíba and to identify possible associated factors with the infections. Two hundred twenty-nine samples from goats and two hundred five from sheep were analyzed by Indirect Immunofluorescence Reaction (IFAT) using a cutoff point of 1:50. The presence of anti-*N. caninum* antibodies was identified in 28.4% (65/ 229; 95% Confidence Interval: 22.6-34.2) of the goat samples and in 12.7% (26/ 205; 95% CI: 8.2 – 17.2) of the sheep samples. Contact between goats and dogs (*Odds ratio* 4.81; CI 1.13 – 2.67; p = 0.041) and cattle (OR. 1.87; CI 1.13 – 2.67; p = 0.002) was identified as a risk factor for goats and contact between sheep and dogs (OR 2.32; CI 1.58 – 3.14; p = 0.026) and history of abortion (OR 1.94; CI 1.28 – 2.90; p = 0.001) was considered a risk factor for sheep. The study revealed a high seroprevalence of anti-*N. caninum* antibodies in slaughtered goats and sheep in Paraíba. Risk factors such as contact with dogs/cattle and abortion history underscore the need for preventive measures to control infection and enhance animal health management.

## Resumo

O objetivo deste estudo foi descrever a soroprevalência de anticorpos anti-*Neospora caninum* em caprinos e ovinos abatidos em abatedouros públicos do Estado da Paraíba, e identificar os possíveis fatores associados às infecções. Foram analisadas 229 amostras de soro de caprinos e 205 de ovinos pela Reação de Imunofluorescência Indireta (RIFI), utilizando-se ponto de corte de 1:50. A presença de anticorpos anti-*Neospora caninum* foi identificada em 28,4% (65/229; Intervalo de confiança 95%: 22,6-34,2) das amostras de caprinos e em 12,7% (26/205; IC 95%: 8,2-17,2%) das amostras de ovinos. O contato de caprinos com cães (*Odds ratio* 4,81; IC 2,07-8,87; p = 0,041) e bovinos (OR. 1,87; IC 1,13 – 2,67; p = 0,002) foram identificados como fatores de risco para caprinos; e o contato de ovinos com cães (OR. 2,32; IC 1,58-3,14; p = 0,026) e o histórico de aborto (OR. 1,94; IC 1,28-2,90; p = 0,001) foram considerados fatores de risco para ovinos. O estudo revelou alta soroprevalência de anticorpos anti-*N. caninum* em caprinos e ovinos abatidos na Paraíba. Fatores de risco, como contato com cães/bovinos e histórico de aborto, destacam a necessidade de medidas preventivas para conter a infecção e melhorar o manejo da saúde animal.

## Introduction

Neosporosis is a disease caused by the protozoan *Neospora caninum*, which has domestic and wild canids as its definitive host (Lindsay et al., 1999; Dubey et al., 2011). As intermediate hosts are species such as sheep, goats, and cattle. Although cattle are considered more susceptible to neosporosis, abortions, placental necrosis, fetal death, stillbirths, and other reproductive problems have also been reported in sheep and goats, although these animals may have some resistance to the disease (Buxton et al., 2002; Mesquita et al., 2018). In small ruminants, *N. caninum* can be transmitted horizontally or vertically, and the association of the two forms of transmission is often identified as a way of perpetuating the disease in herds (Nunes et al., 2017; Pereira et al., 2021; Bezerra et al., 2022a, b).

The occurrence of a high rate of endogenous vertical transmission in naturally infected sheep (6/ 19 – 31.6%) was observed in the semi-arid region of the state of Paraíba, Brazil, in which chronically infected females gave birth to clinically healthy animals, but infected and with potential for transmissibility to future offspring or to the definitive host (Feitosa et al., 2021). Vertical transmission seems to be of greater importance in the perpetuation of the parasite in small ruminants when compared to horizontal transmission, since the same female can transmit the protozoan to several subsequent generations, making the occurrence and maintenance of the disease in the herd possible even in the absence of the definitive host (Pereira et al., 2021).

However, horizontal transmission may be favored due to the eating habits of dogs, which may involve the ingestion of animal’s tissues such as placental remains (Lindsay et al., 1999; Robbe et al., 2016). Contact with dogs increases the risk of infection in small ruminants, although other factors such as a history of abortion, contact with other species, and water supply are also associated with the infection. It is essential to consider these factors when identifying reproductive problems within the herd (Machado et al., 2011; Topazio et al., 2014; Rodrigues et al., 2020). Thus, control and prevention measures should be focused simultaneously on reducing vertical transmission and reducing horizontal transmission.

These facts make diagnosis and regional epidemiological knowledge important tools to better understand the dynamics of neosporosis transmission in herds. In addition, knowledge of seroprevalence and factors associated with *N. caninum* infection is necessary for the development of an effective control and prophylaxis methodology. Current knowledge about the seroprevalence of neosporosis in small ruminants in Brazil is still scarce. Faced with this gap, the objective of this study was to describe the seroprevalence of *N. caninum* in sheep and goats slaughtered in public slaughterhouses in two cities in the semi-arid region of the state of Paraíba and to identify the risk factors related to these infections.

## Material and Methods

### Sampling

To determine the number of samples to be evaluated, simple random sampling was performed according to Thrusfield (2007):


$$n=\frac{Z^{2}\times P\left(1-P\right)}{d^{2}}$$ (1)

n = number of samples

Z = normal distribution value for the confidence level of 95%

P = expected prevalence of 17.4% in goats and 4.7% in sheep (Moraes et al., 2011).

d = sampling error of 5%

**Figure 1:**
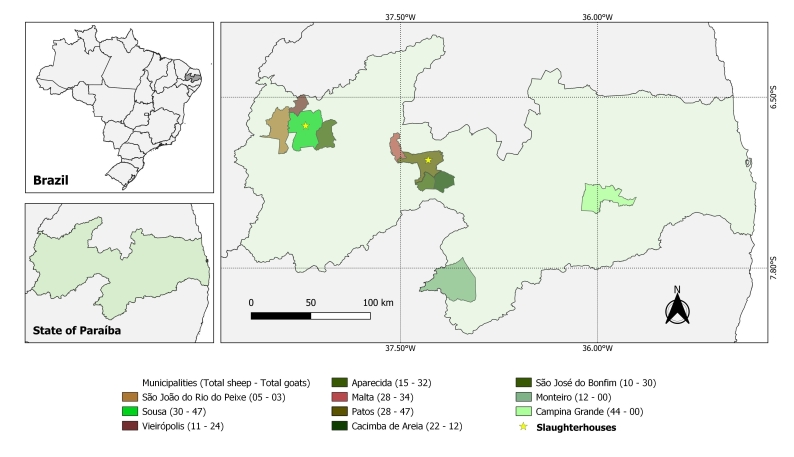
Geographical data pertaining to the source municipalities of goats and sheep, as well as the visited slaughterhouses for sample collection in the state of Paraíba, Brazil.

The minimum number of samples to be evaluated determined by the sample calculation was 220 samples for goats and 69 sheep. However, for safety and due to the availability of animals, staff, and materials, a total of 229 goat samples were collected (106 in Sousa and 123 in Patos), along with 205 sheep samples (105 in Sousa and 100 in Patos). The samples were collected from June 2019 to November 2020. The animals came from 10 municipalities in the semi-arid region of the state of Paraíba (Figure 1).

Blood samples were collected during the slaughter of the animals, at the bleeding, in glass tubes previously identifies and without anticoagulant, to obtain serum. They were stored frozen at -20°C at the Laboratório de Imunologia e Doenças Infectocontagiosas (LIDIC), do Hospital Veterinário do Instituto Federal da Paraíba-IFPB, campus Sousa.

### Serological diagnosis

For the detection of anti-*N. caninum*, the samples were submitted to the Indirect Immunofluorescence Reaction (IFAT) using the 1:50 cutoff point (Lima et al., 2008), following the method described by Camargo (1974), with the Nc-1 tachyzoite strain fixed on a slide as antigen. Serum was diluted using 3μL added to 147μL of PBS pH 7.2. Diluted samples were distributed on slide and incubated in an oven (37°C for 30 minutes). Then, they were submitted to three immersion washes of five minutes each, with PBS pH 7.2. After drying, the slides were covered with 20μL of anti-goat or ovine IgG antibodies conjugated to fluorescein isothiocynate (whole molecule, SIGMA, St. Louis, Mo, USA). Subsequently, they were incubated for 30 minutes at 37°C and washed again with PBS pH 7.2. After drying, buffered glycerin was added to the slides and readings were performed in a fluorescence microscope with ultraviolet light emission, 400x magnification. Samples that showed total peripheral fluorescence of tachyzoites were considered positive, with positive samples submitted to sequential dilutions in base two until negative.

### Epidemiological questionnaire

During the collections, epidemiological questionnaires were applied to the owners. The variables analyzed and their respective categories were: gender (male or female), breed (pure or crossbred), age (< 1 year, 1-2 years, >2 years), rearing system (semi-intensive or extensive), rearing aptitude (meat, dairy or mixed), access to water (treated or untreated), contact with wild animals (yes or no), contact with other domestic animals (cattle, horses, pigs, sheep, goats, dogs, cats or birds), vaccination history (yes- which ones?; or no) and history of abortions (yes or no).

### Statistical analysis

Descriptive statistical analysis was used to calculate the frequencies of the results obtained in the IFAT. To identify the factors associated with the prevalence of anti-*N. caninum*, data from the epidemiological questionnaires were used in two stages, univariate and multivariate analyses. In the univariate analysis, each independent variable was correlated with the dependent variable (seropositivity), and those with a p-value ≤ 0.2, according to the Chi-square test (Zar, 1999), were selected for the multivariate analysis using multiple logistic regression (Hosmer & Lemeshow, 2000), with a significance level of 5%. To verify any possible collinearity between the data, a correlation test was applied and, if the correlation coefficient was greater than 0.9, one of the variables was eliminated using the criterion of biological plausibility. To verify the fit of the model, the Chi-square parameters and the Omnibus test were used. The results were analyzed using GraphPad Prism 8.0.1 Software.

## Results

Among the 229 evaluated goats, 65 (28.4%; 95% CI: 22.6-34.2) were positive for anti-*N. caninum*; in sheep, 12.7% (26/205; 95% CI: 8.2-17.2%) were seropositive. For both species, antibody titers ranged from 1:50 to 1:12,800, with 1:200 being the most frequent titer (Table 1).

**Table 1:** Distribution of anti-*Neospora caninum* antibodies by Indirect Immunofluorescence Reaction (IFAT) in goats and sheep slaughtered in the semi-arid region of the state of Paraíba, Brazil.

Seropositivity in goats									
Titration	1:50	1:100	1:200	1:400	1:800	1:1600	1:3200	1:6400	1:12800
Total (%)	7 (10.8)	13 (20)	19 (29.2)	14 (21.6)	7 (10.8)	3 (4.6)	1 (1.5)	-	1 (1.5)
Seropositivity in sheep									
Titration	1:50	1:100	1:00	1:400	1:800	1:1600	1:3200	1:6400	1:12800
Total (%)	3 (11.5)	3 (11.5)	8 (30.8)	1 (3.9)	2 (7.7)	2 (7.7)	2 (7.7)	3 (11.5)	2 (7.7)

In the univariate analysis, the variables that showed a statistically significant difference (p ≤ 20) and were selected for the multivariate analysis for goats were age, contact with dogs, contact with cattle and history of abortions. For sheep, they were rearing system, contact with dogs, contact with cattle and history of abortion (Table 2).

**Table 2:** Univariate analysis of factors associated with *Neospora caninum* infections in goats and sheep slaughtered in the semi-arid region of the state of Paraíba, Brazil.

Variables/category	Goats			Sheep		
	Total	Positives (%)	*p*	Total	Positives (%)	*p*
Age						
< 1 year	24	4 (16.6)	0.111*	20	2 (10)	0.869
1 – 2 years	37	15 (40.5)		44	5 (11.3)	
> 2 years	168	46 (27.4)		141	19 (13.4)	
Breeding system						
Extensive	75	18 (24)	0.35	29	9 (31)	0.003*
Semi-intensive	154	47 (30.5)		176	17 (9.6)	
Contact with dogs						
Yes	138	46 (33.3)	0.051*	74	15 (20.2)	0.017*
No	91	19 (20.8)		131	11 (8.3)	
Contact with cattle						
Yes	108	37 (34.2)	0.054*	80	15 (18.7)	0.051*
No	121	27 (22.3)		125	11 (8.8)	
History of abortion						
Yes	85	29 (34.1)	0.172*	194	22 (11.3)	0.036*
No	144	36 (25)		11	4 (36.3)	

* Variables that presented p-value ≤ 0.20 by the chi-square or Fisher’s exact test.

The risk factors identified through multivariate analysis for goats were contact with dogs (OR. 4.81; IC 2.07-8.87; p = 0.041) and contact with cattle (OR. 1.87; IC 1.13- 2.67; p = 0.002) and for sheep the variables identified were contact with dogs (OR. 2.32; IC 1.58-3.14; p = 0.026) and history of abortions (OR. 1.94; IC 1.28-2.90; p = 0.001) (Table 3).

**Table 3:** Multivariate analysis of risk factors associated with *Neospora caninum* infections in goas and sheep slaughtered in the semi-arid region of the state of Paraíba, Brazil.

Risk factors	*Odds ratio*	CI 95%	*p*
Goats			
Contact with dogs	4.81	[2.07–8.87]	0.041
Contact with cattle	1.87	[1.13-2.67]	0.002
Sheep			
Contact with dogs	2.32	[1.58-3.14]	0.026
History of abortion	1.94	[1.28-2.90]	0.001

## Discussion

The seroprevalence above 28% for anti-*N. caninum* antibodies in goats found in this study was the highest reported in goats in Brazil until this date, the previously described seroprevalence ranges from 1.05% (Lima et al., 2008) to 26.10% (Braz et al., 2018). In the Northeast region, seroprevalences were found that corroborate with was presented in this study. In the state of Pernambuco, 20.37% (Galvão et al., 2023) and 26.6% (Tembue et al., 2011) of seropositivity were fescribed. In the state of Bahia was identified 15% of seropositivity in goats (Uzêda et al., 2007), and in Paraíba 26.10% (Braz et al., 2018), which demonstrates uniformity in the occurrence of *N. caninum* in goats from different states of this region, due to the similarity in climate and herd management practices. The disparity between the seroprevalence rates reported in a study conducted by Faria et al. (2007) (3.3% - 10/306) in Patos, Paraíba, and the findings of our study is quite substantial. Notably, both studies involved sample collections at the Patos slaughterhouse. However, the temporal gap of over a decade between these studies may imply an upward trend in the prevalence of neosporosis within herds in that particular region.

In other regions of Brazil has been demonstrate the same uniformity between the studies in goats from a same region. In Southeast region Figliuolo et al. (2004a) have identified 6.4% of seroprevalence in the state of São Paulo; Varaschin et al. (2011) and Andrade et al (2013) have identified 10.7% state of Minas Gerais. In South region Topazio et al. (2014) has described 4.58% of seropositivity in Santa Catarina and Romanelli et al. (2020) 6.3% in Paraná. The North region is the only with the highest lack of information about the seroprevalence in goats, the scarcity of data on the occurrence of *N. caninum* in goats from different regions of Brazil is a factor that makes it difficult to compare the distribution of the parasite in the country’s herds (Moraes et al., 2011).

In sheep, the seroprevalence found in this study was above 12%, remaining within the seroprevalence range described in studies from different states and regions of Brazil, ranging from 1.8% (Soares et al., 2009) to 64.2% (Tembue et al., 2011). The result of the present study corroborates the data found by Mendonça et al. (2019) in the state of Sergipe, who obtained seropositivity of 12.4% and by Nunes et al. (2020) with 13% seroprevalence in the state of Alagoas, both in the Northeast region. Despite the similarity of these seroprevalences, Moraes et al. (2011) in the state of Maranhão found seroprevalence of 4.69% in sheep and Tembue et al. (2011) found a seroprevalence of 64.2% in sheep from the state of Pernambuco, both in Northeast region which demonstrate a high divergence in states from the same region where the climatic conditions are similar.

Such variations are also observed in different regions of Brazil. In the Southeastern region, there appears to be consistency in the distribution of the parasite, with seroprevalence rates ranging from 9.2% to 12.8% in the state of São Paulo (Figliuolo et al., 2004b; Langoni et al., 2011; Machado et al., 2011; Cosendey et al., 2018), from 8.1% to 13.1% in the state of Minas Gerais (Salaberry et al., 2010; Andrade et al., 2012), and a reported 6.2% in the state of Rio de Janeiro (Cosendey et al., 2018). In the South region, seroprevalence rates range from 9.5% to 17.6% (Romanelli et al., 2007, 2021), suggesting a possible increasing trend in seropositivity. In the North region, seroprevalence rates range from 13.74% to 60.6% (Guimarães et al., 2015; Maia et al., 2021), a range similar to that observed in the Northeast. These data illustrate a significant divergence from the findings presented in this study and others conducted in Brazil. It is important to acknowledge that variations between epidemiological studies can be attributed to several factors, including the inherent climatic conditions of the studied regions, the age of animals, the size of herds, the types of management practices, and variations in laboratory procedures such as evaluation methods, cutoff points used, the experience of the evaluator, and equipment maintenance (Dubey et al., 2007; Nunes et al., 2020).

The risk factors identified in the multivariate analysis for goats were contact with dogs and contact with cattle. Contact with dogs increased the chances of infection by 4.81 time according to the odds ratio. In according to the systematic review and meta-analysis performed by Rodrigues et al. (2020), contact with dogs is the most frequently reported risk factors associated with *N. caninum* infection in goats. Dogs are the definitive hosts and their presence on the properties allows the occurrence of horizontal transmission associated with vertical transmission, which facilitates the propagations and perpetuation of the parasite in the herd (Nunes et al., 2017; Pereira et al., 2021).

Contact with cattle increased the chances of infection by 1.87 times according to the Odds ratio, probably because the parasite is an important cause of abortion for cattle, which favors the transmission of the disease to the definitive hosts, due to the ingestion of placental remains and allows the occurrence of the two main forms of transmission and the transmission to goats on properties that carry out goat and cattle farming concomitantly (Dubey et al., 2007; Robbe et al., 2016).

In sheep, contact with dogs and history of abortion were considered risk factors. The contact of sheep with dogs increased the chances of infection by *N. caninum* by 2.32 times. Despite the presence of the definitive host enabling the occurrence of horizontal and vertical transmission, few studies have identified contact with dogs as a risk factor for sheep in Brazil (Machado et al., 2011; Gheller et al., 2016;). Furthermore, Mendonça et al. (2019) considered the presence of dogs on the property as protective factor for sheep, since the presence of dogs can limit the access of other canids and reduce pasture contamination. This divergence between studies of risk factors may occur due to the lack of standardization of epidemiological questionnaires, as well as the methodologies applied in epidemiological surveys (Dubey et al., 2007).

The presence of a history of abortion on the property increased the chances of infection by 1.94 times. The history of abortion was also identified as a risk factor in other studies (Machado et al., 2011; Gheller et al., 2016). Abortion in pastures or open areas facilitates ingestion of the placenta by dogs, which favors horizontal transmission through contamination of pastures with dog feces and may increase the occurrence of neosporosis in the herd (Romanelli et al., 2021). In addition, females are usually slaughtered older than males, which leads to a longer exposure time to the possibility of infection by *N. caninum* and allows the occurrence of congenital transmission more than once in a breeding female leading to contamination of several generations and greater probability of occurrence of abortions, for this reason *N. caninum* must be considered whenever there are cases of abortions, stillbirths and weak fetuses in the herd (Nunes et al., 2017; Pereira et al., 2021).

## Conclusion

The seroprevalence of *N. caninum* found in this study in goats was the highest ever recorded in Brazil so far and indicates an increase in cases of infection by the parasite in the state of Paraíba. Contact between goats and dogs and cattle has been identified as a risk factor and should be avoided. In sheep, seroprevalence was considered high and contact between sheep and dogs was considered a risk factor. As well as the history of abortion in sheep, which should be considered and indicator of the disease in the herd. Understanding the epidemiology of the disease is essential to establish effective control and prophylaxis measures and minimize economic losses resulting from reproductive problems.
